# H_2_S, a novel gasotransmitter, involves in gastric accommodation

**DOI:** 10.1038/srep16086

**Published:** 2015-11-04

**Authors:** Ailin Xiao, Hongjuan Wang, Xin Lu, Jianchun Zhu, Di Huang, Tonghui Xu, Jianqiang Guo, Chuanyong Liu, Jingxin Li

**Affiliations:** 1 Department of Physiology, Shandong University School of Medicine, Jinan, People’s Republic of China; 2 Department of gastroenterology, Second Hospital, Shandong University, Jinan, People’s Republic of China

## Abstract

H_2_S is produced mainly by two enzymes:cystathionine-β-synthase (CBS) and cystathionine-γ-lyase (CSE), using L-cysteine (L-Cys) as the substrate. In this study, we investigated the role of H_2_S in gastric accommodation using CBS^+/−^ mice, immunohistochemistry, immunoblot, methylene blue assay, intragastric pressure (IGP) recording and electrical field stimulation (EFS). Mouse gastric fundus expressed H_2_S-generating enzymes (CBS and CSE) and generated detectable amounts of H_2_S. The H_2_S donor, NaHS or L-Cys, caused a relaxation in either gastric fundus or body. The gastric compliance was significantly increased in the presence of L-Cys (1 mM). On the contrary, AOAA, an inhibitor for CBS, largely inhibited gastric compliance. Consistently, CBS^+/−^ mice shows a lower gastric compliance. However, PAG, a CSE inhibitor, had no effect on gastric compliances. L-Cys enhances the non-adrenergic, non-cholinergic (NANC) relaxation of fundus strips, but AOAA reduces the magnitude of relaxations to EFS. Notably, the expression level of CBS but not CSE protein was elevated after feeding. Consistently, the production of H_2_S was also increased after feeding in mice gastric fundus. In addition, AOAA largely reduced food intake and body weight in mice. Furthermore, a metabolic aberration of H_2_S was found in patients with functional dyspepsia (FD). In conclusion, endogenous H_2_S, a novel gasotransmitter, involves in gastric accommodation.

The stomach has variety of functions including reservoir functions. Disorders of the reservoir functions result in symptoms of early satiety and anorexia, which are the major symptoms of patients with functional dyspepsia (FD). Gastric accommodation consists of two types of relaxation: the receptive relaxation and the adaptive relaxation. These physiological responses are important to accommodate the intake of food and liquid. Adaptive relaxation is a reflex in which the fundus of the stomach dilates in response to small increases in intragastric pressure when food enters the stomach. Receptive relaxation is a reflex in which the gastric fundus dilates when food passes down the pharynx and the esophagus.

Some gastrointestinal hormones and chemical mediators such as gastrin, histamine^1^, serotonin, vasoactive intestinal peptide (VIP)^2^ and nitric oxide (NO)^3^,^4^,^5^ have been shown to mediate these two types of relaxations.

In the gastrointestinal tract, NO is an important non-adrenergic, non-cholinergic (NANC) inhibitory neurotransmitter which is released in response to nerve stimulation and relaxes smooth muscles^6^,^7^. Animal studies have consistently shown that basal tone is decreased by vagal stimulation and that this effect is blocked by the NO inhibitor^8^,^9^,^10^,^11^. Besides NO and CO, hydrogen sulfide (H_2_S) is the third gasotransmitter. H_2_S is produced mainly by two enzymes:cystathionine–β–synthase (CBS) and cystathionine-γ–lyase (CSE), using L-cysteine (L-Cys) as the substrate^12^,^13^,^14^. CBS and CSE are expressed in the enteric nervous system (ENS)^15^. In the gastrointestinal tract, sodium hydrogen sulfide (NaHS), a source of H_2_S, can reduce spontaneous or acetylcholine (ACh)-induced contraction of ileal smooth muscles^16^,^17^. H_2_S also causes concentration-dependent relaxation of pre-contracted smooth muscles in the mouse gastric fundus and distal colon^18^,^19^. Muscle contractions of the mouse colon and jejunum were also inhibited by application of NaHS^20^. H_2_S is similar with the two kinds of endogenous gas signal molecules of CO and NO, they are very important bio-regulating substances, and share some common characteristics. We hypothesize that beside NO, H_2_S is another gasotransmitter which involves in the mechanical accommodation of the stomach. In the present study, we therefore examined the role of H_2_S in receptive and adaptive relaxation of the mouse stomach.

## Materials and Methods

### Animals

Male BLAB/c mice weighing 35–45 g, kept in individual cages with raised mesh bottoms, were deprived of food but allowed free access to tap water for 18 hr before the experiments. Animals were sacrificed by cervical dislocation and the stomach was quickly removed and placed into aerated (5% CO_2_ and 95% O_2_) Krebs solution. Wild-type (WT) and CBS^+/−^ mice on C57BL/6J background were obtained from the Jackson Laboratory (BarHarbor, ME). All experimental procedures were conducted in accordance with the Guidelines for the Care and Use of Laboratory Animals of Shandong University, and the present study was approved by the Experimental Animal Research Committee of Shandong University China (number ECAESDUSM 2012029).

### Western blots

Gastric biopsy specimens were obtained from 8 patients with FD fulfilling the Rome III criteria and 7 healthy volunteers. Biopsy samples were taken for western blot detection. Informed consent was obtained from each patient and approval granted from the Medical Ethics Committees of Shandong University (number MECSDUMS 2013023). Tissue was homogenized in ice-cold lysis buffer. The ice-cold lysis buffer contained: 50 mM Tris (pH 7.4), 150 mM NaCl, 1%TritonX-100, 1% sodium deoxycholate, 0.1% SDS, 1 mM NaF, 1 mM Na_3_VO_4_, 1 mM EDTA and 0.5 μg/ml leupeptin. After centrifugation, the supernatant was boiled for 10 min. Ten to thirty mg of denatured proteins were separated on 10% SDS polyacrylamide gels and then transferred to a PVDF membrane. Membranes were blocked for one hour using 5% non-fat dry milk in Tris-buffered saline with 0.05% Tween-20, then washed in Tween-Tris-buffered saline (0.1% Tween 20, 50 mM Tris and 150 mM NaCl), followed by overnight incubation at 4 °C with a rabbit polyclonal CBS antibody (Santa Cruz Biotechnology, Santa Cruz, CA, 1:1000 dilution) or a rabbit polyclonal CSE antibody (Abcam, Cambridge, UK, 1:1000 dilution). Membranes were washed in Tween-Tris-buffered saline and incubated with an anti-horseradish-peroxidase conjugated secondary antibody (ZSGB biology, Beijing, China, 1:20000) for one hour. The membranes were washed again and exposed to ECL. The blot films were scanned, and the band densities were calculated using the Quantity One analysis software (Bio-Rad). The values of blot densities were normalized to the levels of respective β-actin blots.

### Immunofluorescence

Paraffin sections were roasted 90 min at 65 °C, dewaxed in xylene twice for 10 min and then rehydrated in 100, 100, 95, 95, 90 and 80% of ethanol and running tap water for 5 min each, in order. Tissue sections underwent antigen retrieval in a solution consisting of 0.01 M citrate and 0.01 M sodium citrate before they were blocked in PBS containing 10% goat serum for 1 h at room temperature. The sections were incubated with a polyclonal CBS antibody (Santa Cruz Biotechnology, Santa Cruz, CA, 1:100 dilution) or polyclonal CSE antibody (Abcam, Cambridge, UK, 1:100 dilution) overnight at 4 °C. After being washed in PBS, the sections were incubated for 1 h with Alexa Fluor 568 goat anti-rabbit IgG (HtL) (1: 600; Invitrogen Carlsbad, CA, USA) at room temperature. The sections were washed again and incubated in DAPI (1:1,000) for 10 min at room temperature. In negative controls, the sections were incubated with PBS instead of the primary antibody. We repeated the immunohistochemistry of each protein in eight tissue slices of four samples. The fluorescence intensity for a specific protein stain was set below the threshold for the negative control.

### The release of H_2_S in fundus

Tissues were homogenized in 50 mM ice-cold potassium phosphate buffer pH 6.8. The reaction mixture contained (mM): 10% (w/v) tissue homogenate (0.5 ml), 100 mM potassium phosphate buffer (pH = 7.4, 0.5 ml), 20 mM pyridoxal 5′-phosphate (0.1 ml) and 10 mM L-Cys (0.1 ml). The reaction was performed in a 25-ml flask containing the reaction mixture. Before being sealed, the flask was flushed with N2. The reaction was initiated by transferring the flasks from ice to a 37 °C shaking water bath. After incubation at 37 °C for 90 min, trichloroacetic acid (50%, 0.5 ml) was added to the reaction mixture to stop the reaction and incubated at 37 °C for an additional 60 min. The contents were then transferred to test tubes, each containing 3.5 ml of ultra-pure water. Subsequently, 0.5 ml of 20 mM N,N-dimethyl-p-phenylenediamine sulphate in 7.2 M HCl was added, immediately followed by the addition of 0.4 ml 30 mM FeCl3. After 20 min of incubation at room temperature, the optical absorbance of the resulting solutions was measured at 670 nm. A standard curve was generated with known concentrations of NaHS. The H_2_S concentration was calculated against the calibration curve of the standard H_2_S solutions.

### Muscle tension experiment

The fundic portion of the stomach was dissected free. One full wall thickness fundus strips (2 × 10 mm) were prepared by cutting in the direction of the longitudinal muscle layer. After a silk thread (USP 4/0) was attached to both ends of the strips, they were mounted in 7 ml organ baths. One end of each strip was fixed, while the other was connected to a force displacement transducer for continuous recording of isometric tension. After an equilibration period of 30 min with flushing every 10 min at a load of 1 g (±0.2 g), the length-tension relationship was determined. Strips were subsequently incubated with NaHS (10^−3^ mol/L) and determine the tension.

### Recordings of intragastric pressure *in vivo* (IGP)

Mice were anesthetized in urethane (25%, 1.5 g/kg, ip). A homemade balloon (maximum volume 1.5 ml) attached to pressure sensor was inserted into the bottom of the stomach from incision on anterior wall of duodenal bulb. The volume of balloon was increased stepwise from 0.1 to 0.3, 0.5 ml by injection water through T-branch pipe. The pressure inside balloon increased sharply and then slowly lower to reach a platform due to the relaxation of the bottom of the stomach. IGP was recorded and viewed in real time using customized PowerLab Chart 5 v5.1 software (AD Instruments). IGPs were set at 0 mmHg and recorded in response to stepwise isovolumetric distensions. Gastric adaptive relaxation compliance expressed as the rate of decline of IGPs to each volume stimuli (0–20 s) and plateau pressure expressed as plateau values minus basal values were evaluated using the same software. Responses with or without pretreatment with L-Cys, AOAA, PAG, SAM and NaHS were evaluated.

### The NANC relaxation of fundus strips induced by electrical field stimulation (EFS)

The fundic portion of the stomach was dissected free. One full wall thickness fundus strips (2 × 10 mm) were prepared by cutting in the direction of the longitudinal muscle layer. Muscle strips were mounted in 7 ml double-jacketed organ baths containing Krebs solution, gassed with 95% O_2_–5% CO_2_ mixture. Prewarmed water (37 °C) was circulated through the outer jacket of the tissue bath via a constant-temperature circulator pump. One end of each strip was fixed, while the other was connected to a force displacement transducer for continuous recording of isometric tension. EFS was applied via two platinum electrodes (6 mm apart). All experiments were performed at optimal load. Therefore, after an equilibration period of 30 min with flushing every 10 min at a load of 1.5 g (±0.2 g), the length-tension relationship was determined. To investigate the effects of H_2_S on NANC relaxant responses, isoproterenol (1 μM), atropine (2 μM) were added to the bath medium, to rule out the adrenergic and the cholinergic influences, respectively. Each fundus strip was allowed to equilibrate for at least 30 min before 5-HT (0.5 μM) was added to produce a sustained increase. After a further 10-min equilibration period, the responses to electrical field stimulation (EFS; 80 V, 0.5 ms, 4–8–16 Hz for 15 s with a 2 min interval) were obtained in the presence or absence of L-Cys (1 mM) or AOAA (1 mM).

### Effects of H_2_S signal pathway on food intake

Mice were randomly divided into six groups. Abdominal cavity injection was performed every 48 hours with normal saline (10 ml/kg), L-Cys (50 mg/kg), AOAA (50 mg/kg), PAG (100 mg/kg), SAM (50 mg/kg) and NaHS (5 mg/kg), respectively, from 0 day to 16 day. In this period, average food intake, water intake and body weight of each group were measured every 24 hours.

### Solutions and drugs

Krebs solution was a buffer solution containing (mmol/L): NaCl 120.6, KCl 5.9, CaCl_2_ 2.5, NaH_2_ PO_4_ 1.2, MgCl_2_ 1.2, NaHCO_3_ 15.4 and glucose 11.5. PH was 7.4. For immunohistochemical experiments, phosphate buffered saline (PBS) was used containing (mmol/L):NaCl 135, KCl 2.7, KH_2_PO_4_ 1.5, and K_2_HPO_4_ 8, pH was 7.4. For western blot experiments, Tris-HCL buffered saline (TBS) was used containing (mmol/L):Tris 50, NaCl 150. pH was adjusted to 7.4 with HCl.

L-Cys, NaHS, AOAA, SAM and PAG were from Sigma. Pyridoxal 5-phosphate and dimethyl aniline hydrochloride (DMPD) were from Aladdin (Shanghai, China). Zinc acetate, FeC l3 and trichloroacetic acid were from Damao chemical reagent company (Tianjin, China). If not indicated specially, the drugs were from chemical reagent co., LTD of national medicine bloc (Shanghai, China).

### Statistical analysis

The data are presented as means ± standard error of the mean (SEM), n is the number of tissues examined. The Student’s t-test was used for comparison between the two sets of data, and ANOVA analysis was used for group comparison. A *P* < 0.05 was considered statistically significant.

## Results

### Western Blot and immunoflurence studies

**Figure 1:**
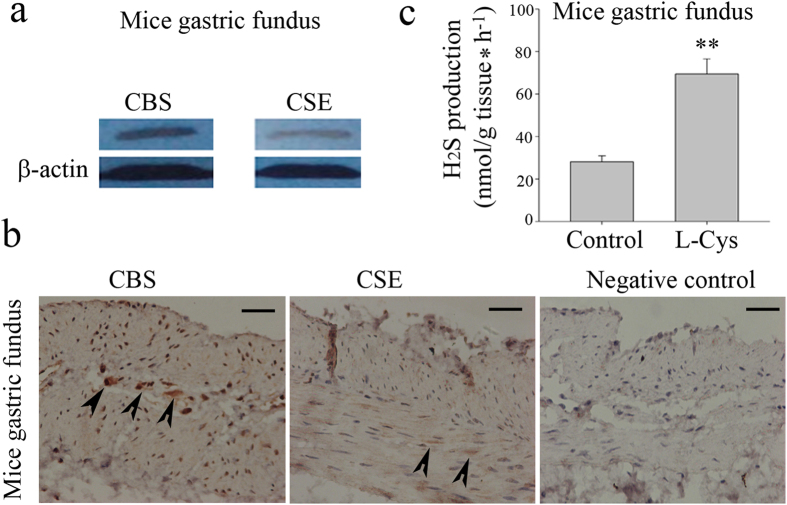
Mice gastric fundus expressed CBS and CSE and generated detectable amounts of H_2_S. CBS and CSE were expressed in mice fundus (**a**). CBS was expressed on the soma of the myenteric neurons of gastric fundus muscle myenteric plexus. Bundles of muscular tissue showed a clear immunoreactivity for CSE (**b**). The gastric fundus of mouse generated detectable amounts of H_2_S. The biosynthesis of H_2_S was increased by 3- fold over basal values after incubation of tissue homogenates with L-Cys, the CBS/CSE substrate (**c**). Scale bar: 50 μm. n = 8; **P < 0.01.

CBS and CSE were expressed in mice fundus as demonstrated by Western blot studies (Fig. 1a). The immunohistochemistry study shows that CBS was expressed on the soma of the myenteric neurons of gastric fundus muscle myenteric plexus (Fig. 1b). In addition, bundles of muscular tissue showed a clear immunoreactivity for CSE (Fig. 1b).

### H_2_S Production in gastric fundus

The gastric fundus of mouse generated detectable amounts of H_2_S (Fig. 1c). The biosynthesis of H_2_S was increased by 3- fold over basal values after incubation of tissue homogenates with L-Cys, the CBS/CSE substrate (Fig. 1c). Therefore, gastric fundus is capable of synthesizing H_2_S from L-Cys.

### Effect of H_2_S donor, NaHS or L-Cys in gastric fundus strips

**Figure 2:**
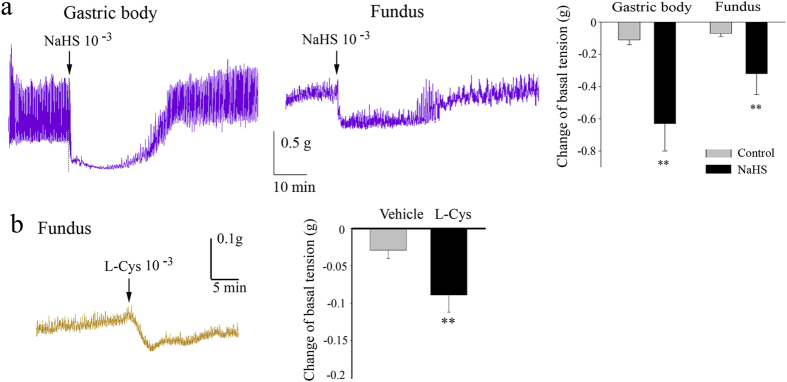
Effect of H_2_S donor, NaHS or L-Cys in gastric fundus Strips. Representative recordings of the effects of NaHS or L-Cys on the contraction of gastric body and fundus muscle strips of mouse. The H_2_S donor, NaHS (**a**) or L-Cys (**b**) caused a relaxation in either gastric body or fundus (**b**). n = 8–10; **P < 0.01.

The gastric fundus or body strips from mice showed spontaneous contraction (Fig. 2). Exogenous H_2_S donor, NaHS (1 mM) caused a relaxation in either gastric body or fundus (Fig. 2a). L-Cys (1 mM), a substrate of CBS/CSE, also induced an inhibition of contractile activity in mice gastric fundus (Fig. 2b).

### Effect of H_2_S signal pathway on IGP *in vivo*

We hypothesized that CBS expressed in inhibitory motor neurons of the gastric myenteric plexus may detect changes in IGP and enhance gastric compliance.

**Figure 3:**
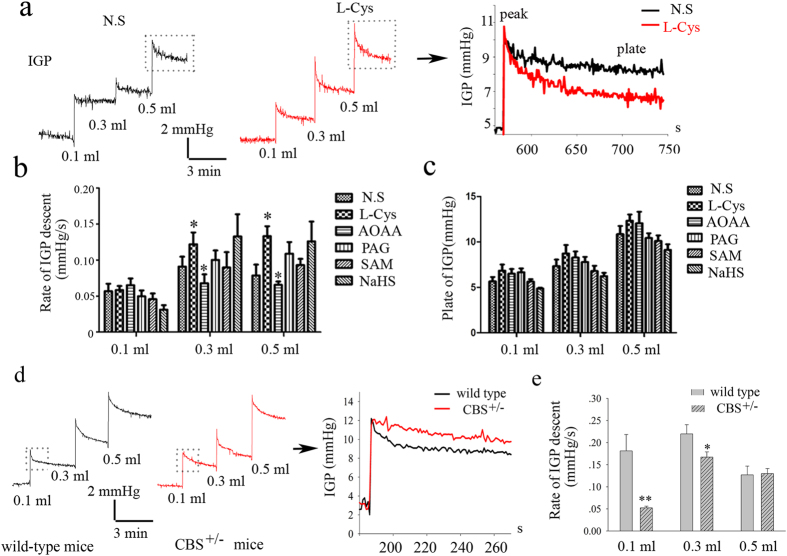
Effect of H_2_S signal pathway on IGP *in vivo*. Representative recordings of the effects of L-Cys on IGP (**a**).The rate of IGP decrease was significantly larger upon pretreatment with the L-Cys (1 mM) than that of control group, which was reduced by AOAA, an inhibitor for CBS (**b**). Plateau IGPs were not affected by pretreatment with either L-Cys or AOAA (**c**). Notably, either exogenous H_2_S donor NaHS or CSE inhibitor PAG had no effect on IGP. Furthermore, the descent rate of IGP was lower in CBS^+/−^ mice than that of littermate wild-type mice (**d**,**e**). Rate of IGP descent: the rate of pressure decrease within 20 s. Plate of IGP: difference between plateau pressure and basal pressure. n = 4–6; *P < 0.05; **P < 0.01.

To test this hypothesis, we measured changes in IGP of mouse stomach responding to volume stimuli *in vivo* in the presence of L-Cys, a substrate of CBS and CSE, or AOAA, an inhibitor for CBS. The results showed that the descent rate of IGP (reflecting gastric compliance) was significantly increased in the presence of L-Cys (1 mM) (Fig. 3a,b). On the contrary, AOAA largely inhibited gastric compliance. However, plateau IGPs were not affected by pretreatment with either L-Cys or AOAA. Notably, exogenous H_2_S donor NaHS, CSE inhibitor PAG or CBS activator SAM had no effect on gastric compliances (Fig. 3c). To confirm the roles of endogenous H_2_S, the CBS knocked out mice was used. The present result showed that the gastric compliance was lower in CBS^+/−^ mice than that of littermate wild-type mice (Fig. 3d,e).

### Responses to NANC nerve stimulation

**Figure 4:**
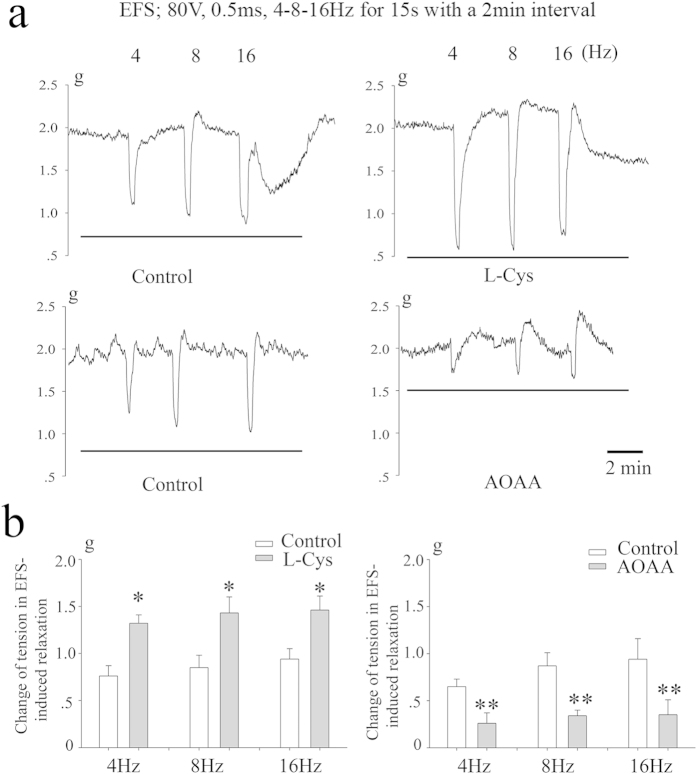
Responses to NANC nerve stimulation. Representative traces of the effects of L-Cys or AOAA on EFS-induced relaxation in fundus muscle strips of mouse (**a**). EFS (4–16 Hz, 80 V, 0.5 ms, 15-s train, 2 min intervals) produced rapid, frequency-dependent relaxations. Relaxant responses of gastric fundus to EFS were significantly enhanced following exposure to L-Cys. However, following incubation with AOAA, the magnitude of relaxations to EFS was greatly reduced (**b**). n = 11; *P < 0.05; **P < 0.01 vs control group.

EFS (4–16 Hz, 80V, 0.5 ms, 15-s train) produced rapid, frequency-dependent relaxations (Fig. 4). Relaxant responses of gastric fundus muscle strips to EFS were significantly enhanced following exposure to L-Cys. However, following incubation with AOAA, the magnitude of relaxations to EFS was greatly reduced (Fig. 4).

### Effect of feeding on the expression of the H_2_S-producing enzyme in mice fundus

**Figure 5:**
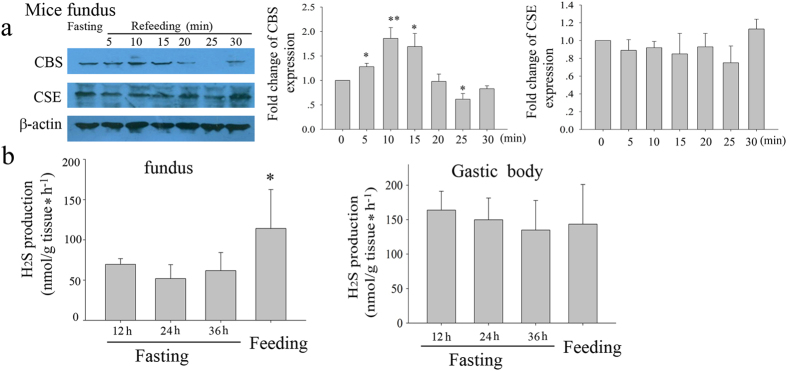
Effect of feeding on the expression of CBS and CSE in mice fundus. The expression of CBS was elevated at 5 min after feeding, and then be back to normal before at 20 min after feeding. However, another H_2_S-generating enzyme CSE is not changed after feeding (**a**). The production of H_2_S was also increased after feeding in mice gastric fundus, but not gastric body (**b**). n = 5; *P < 0.05; **P < 0.01 vs 0 (**a**) or 12 h of fasting (**b**).

The expression of CBS protein was elevated at 5 min and peak at 10 min after feeding, and then be back at 20 min after feeding. However, another H_2_S-generating enzyme CSE is not changed after feeding. The production of H_2_S was also increased after feeding in mice gastric fundus, but not gastric body (Fig. 5).

### Effects of H_2_S signal pathway on food intake and body weight

**Figure 6:**
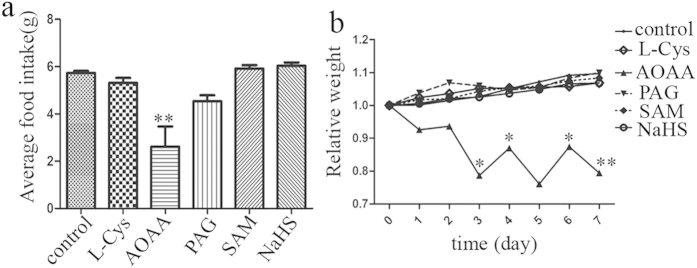
Effects of H_2_S signal pathway on food intake and body weight. Food intake and body weight were largely reduced after injecting AOAA. n = 8; *P < 0.05; **P < 0.01 vs control group (**a**) or the corresponding time point of control group (**b**).

Because H_2_S involves in regulation of IGP, it is possible that H_2_S signal may affect the food intake and body weight. To test this hypothesis, we measured changes in food intake and body weight of mouse after injecting ip L-Cys, AOAA, PAG, SAM and NaHS, respectively. Food intake and body weight were significantly reduced after injecting AOAA (n = 8). However, either L-Cys or NaHS, both H_2_S donors, had no effect on food intake and body weight in mouse. CSE inhibitor PAG or CBS activator SAM also did not affect food intake and body weight, which is consistent with the results of gastric compliances assay (Fig. 6).

### Dysregulation of H_2_S production in FD patients

**Figure 7:**
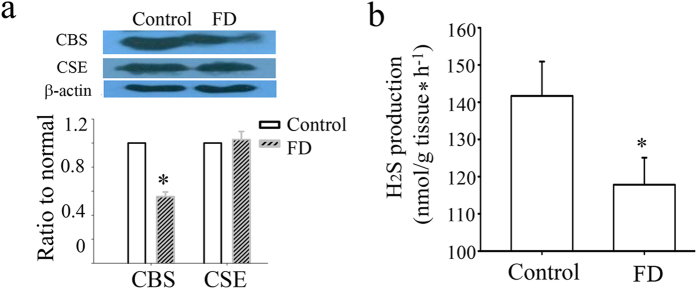
An aberration in H_2_S signal pathway in FD patients. The expression of CBS is downregulated in gastric biopsy sample taken from patients with FD compared with healthy volunteers. However, the expression of CSE, another H_2_S generating enzyme, is not changed (**a**). H_2_S production also was decreased in FD patients (**b**). n = 7–8; *P < 0.05 vs control group.

The expression of CBS protein is downregulated in gastric biopsy sample taken from patients with FD compared with healthy volunteers. However, the expression of CSE, another H_2_S generating enzyme, is not changed (Fig. 7a). By the enzymatic H_2_S production assays, we further confirmed the downregulation of H_2_S production in FD patients (Fig. 7b).

## Discussion

H_2_S has been considered as the third biological gasotransmitter along with NO and CO. Two H_2_S generating enzymes-CBS and CSE have been identified in mammalian systems. CBS and CSE have been documented to be expressed in certain neurons of the mouse^21^, rat^22^, guinea-pig and human enteric nervous systems^15^. In our present study, we demonstrated that both H_2_S generating enzymes also exist in gastric fundus from mouse or human. Moreover, the gastric fundus is capable of synthesizing H_2_S indicated by enzyme activity assay. Growing studies demonstrated that exogenous H_2_S relaxes the gastrointestinal smooth muscle^16^,^17^. H_2_S has an inhibitory role on spontaneous and agonist-mediated rhythmic contractile activity. In isolated ICC of the mouse small intestine, H_2_S inhibits pacemaker activity in ICC^23^ and interacts with nitric oxide in regulating functional pacemaker activity^24^. An endogenous H_2_S contributes to resting membrane potential and spontaneous contractions in the rat colon^25^. However, there are studies that have demonstrated that NaHS at low concentrations increased basal tension in the gastric antrum *in vitro*^26^,^27^ and enhances the gastric emptying *in vivo*^28^. Whether H_2_S is excitatory or inhibitory on gastrointestinal smooth muscle is dependent upon the concentration, regions and species.

In our study, we found that NaHS (1 mM) largely decrease the smooth muscle contraction in the gastric fundus from mouse. Notably, L-Cys, an endogenous H_2_S donor, also exert an inhibitory effect on the spontaneous contraction of gastric fundus smooth muscle in mice. These results suggest that L-Cys/H_2_S regulates the spontaneous contraction of gastric fundus smooth muscle. Recent studies have begun to reveal that H_2_S interacts with NO^29^. H_2_S induces phosphorylation of eNOS and also prevents its degradation^30^,^31^,^32^. CSE knockout mice exhibits dysfunctional eNOS and diminished NO levels, which can be restored by acute H_2_S therapy^30^,^31^. Similarly, CBS (−/+) mice exhibits impaired vascular functions^33^, which is caused by decreased eNOS activity and bioavailability of NO^34^,^35^. H_2_S selectively restored chronic ischemic tissue function and viability by enhancing NO production involving both endothelial NO synthase and sulfide-dependent nitrite reduction mechanisms^36^. However, some studies indicate that H_2_S downregulates the expression of NOS and inhibits the production of NO^37^,^38^,^39^. In our study, L-NAME, a NOS inhibitor, did not influence the L-Cys-evoked relaxation of mice gastric fundus smooth muscle, suggesting that this effect does not depend on NO signaling pathways (data not shown).

The receptive relaxation in response to gastric distention provides an appropriate gastric reservoir for food and enables the stomach to increase the intraluminal volume without rise in the intragastric pressure. In the present study, we observed that the adaptive relaxation induced by gastric distention was enhanced by L-Cys, while inhibited by AOAA *in vivo*. The experiment using CBS knocked out mice further confirmed CBS-derived H_2_S involves the receptive relaxation in response to gastric distention. We further demonstrated in this study that the NANC relaxation of fundus strips induced by EFS was significantly enhanced by L-Cys. In contrast, AOAA attenuated the EFS-induced relaxation of fundus strips, suggesting that endogenous H_2_S may also be involved in the basal receptive relaxation. In addition, the evidences that the expression of CBS and the production of H_2_S in mouse gastric fundus was significantly elevated after feeding further confirm the involvement of endogenous H_2_S in the receptive relaxation.

In FD patients, gastric accommodation is impaired^40^,^41^,^42^. Although impaired gastric accommodation is considered an important pathophysiological mechanism in the development of FD, surprisingly little is known about the aetiology of impaired gastric accommodation. In our study, we found that the H_2_S production was abnormal in FD patients, suggesting that dysregulation of H_2_S production may contribute to FD.

In conclusion, we demonstrate for the first time to our knowledge that a functional H_2_S signal system exists in gastric fundus, and the endogenous H_2_S regulates the gastric accommodation of mouse. The present study suggest the modification of CBS-derived H_2_S pathway is a useful alternative strategy for the treatment of FD-related gastrointestinal disorders.

## Significance of this study

### What is already known on this subject?

- Hydrogen sulfide (H_2_S) is the third gasotransmitter besides NO and CO.
- Two kind of H_2_S-generating enzymes CBS and CSE are expressed in the enteric nervous system (ENS).
- H_2_S regulates gastrointestinal motility.

### What are the new findings?

- Here we reveal that beside NO, H_2_S, another gasotransmitter, involves in gastric accommodation.
- H_2_S is a regulator of gastric accommodation.
- CBS-derived H_2_S involves in receptive and adaptive relaxation of the mouse stomach.
- A metabolic aberration of H_2_S was found in patients with functional dyspepsia (FD).

### How might it impact on clinical practice in the foreseeable future?

Detection of plasma H_2_S concentration may serve as a biomarker in cancer in patients patients with FD. Importantly, this work reveals a potential novel way to treat FD.

## Additional Information

**How to cite this article**: Xiao, A. *et al.* H_2_S, a novel gasotransmitter, involves in gastric accommodation. *Sci. Rep.* **5**, 16086; doi: 10.1038/srep16086 (2015).
